# Protocol for a new family history of addiction density score to aid in the treatment of alcohol and substance use disorders

**DOI:** 10.1016/j.dadr.2025.100321

**Published:** 2025-02-20

**Authors:** Jessica L. Bourdon, Jordan Wright, Sabrina Verdecanna, Mer W. Francis, Vivia V. McCutcheon

**Affiliations:** a Department of Psychology, University of Richmond, 114 UR Drive, Richmond, VA 23173, USA; b Center for Addiction Science, Wellbridge Addiction Treatment and Research, 525 Jan Way, Calverton, NY 11933, USA; c School of Social Work, Virginia Commonwealth University,1000 Floyd Avenue, Box 842027,Richmond,VA 23284,USA; d Department of Psychiatry, Washington University School of Medicine,4560 Clayton Avenue,St. Louis,MO 63110,USA

**Keywords:** Addiction, Genetics, Family history, Density score, Protocol

## Abstract

**Background:**

While molecular and non-molecular genetic testing are the gold standard for assessing a person’s familial liability for substance use disorders, such testing is often inaccessible. Family history information collected at intake is an alternative, but tools to effectively utilize this information are excessively complex. The aims of the study are threefold: 1) Describe a protocol for the collection of family history in a thorough and straightforward manner. 2) Provide an algorithm to convert family history information to numerical scores. 3) Present the aggregated results from the pilot testing of the protocol.

**Methods:**

All patients (*N* = 871) underwent a comprehensive assessment that included the family history protocol. Descriptive statistics, *t-*tests and Pearson Correlation were used to analyze the scores and determine key differences by demographic categories (sex/race/ethnicity/substance/age).

**Results:**

The protocol asked patients four key questions about 1st and 2nd degree relatives while completing a family pedigree. Answers were transferred into an algorithm to output a score for each patient. This score took affectedness and relatedness of each family member into account. The average number of affected relatives was 5.24 (SD=3.17), and there were significant sex, race, and primary substance score differences.

**Conclusions:**

This study provides the addiction field with a novel, freely available, and easily implementable family history protocol that has several potential clinical applications. While more research is needed, pilot results provide a valuable research tool, insight into a typical family history for those at an inpatient addiction treatment center, and steps toward closing the research-to-practice gap in this field.

## Highlights

• Most assessments of genetic liability for substance use are inaccessible and/or cumbersome.
• Properly leveraging family history can have potential clinical applications.
• The Family Density Score offers a simple and easily implemented tool.
• This study freely provides the protocol, algorithm, and pilot data for the Family Density Score.

## Introduction

Proper utilization of genetic information in behavioral healthcare is lacking (Dick, 2018, Doerr and Teng, 2012, Orlando et al., 2011, Bourdon et al., 2020a, Bourdon et al., 2020b). It has been noted for decades that family history is an easier, cheaper, and more easily translatable alternative for a variety of chronic and behavioral disorders (Kendler et al., 1991, Szczerbiński et al., 2019, Andreasen et al., 1977). Specific to AUD/SUD, family history is a robust predictor of recurrent risk of the disorder and has the greatest potential for clinical application for individualized care, though uptake in clinical settings remains poor (Cuijpers and Smit, 2001, Doerr and Teng, 2012, Kendler et al., 2018, Kendler et al., 2003, Khoddam et al., 2015, Bourdon et al., 2020b). For example, children of parents with AUD have 3x elevated risk of developing AUD themselves compared to those whose parents do not have AUD (Kendler et al., 2018). The purpose of this paper is to present a novel, easy-to-use tool for assessing family density of alcohol and substance use in patients that utilizes a pedigree / genogram visual style of collecting family history, then transposing that information into an algorithm that presents patients with a Family Density Score for AUD/SUD. Such a tool could be used in inpatient or outpatient addiction treatment centers to inform clinical decisions about care and as a prevention education tool for those at high risk for SUDs.

### 1.1 Utilization of family history

Family history information is commonly collected as part of medical intake procedures and is always being improved upon (Orlando et al., 2011, Wu et al., 2015, Arar et al., 2013). Outside of medical settings, though, tools that utilize family history and family density for AUD/SUD and other mental health conditions have historically been overly complex and often require querying entire families (Johnson et al., 2019, Kendler et al., 2021a, Khoddam et al., 2015, McCutcheon et al., 2017, Nurnberger et al., 2004, Pandey et al., 2020, Schepis et al., 2023, Kendler et al., 2021b, Kendler et al., 1991). Shorter family history tools are overly reliant on complex diagnostic information and go beyond AUD/SUD (e.g., FHAM, Janca et al., 1994; FH-RDC, Andreasen et al., 1977; Andreasen et al., 1986). This is cumbersome and not realistic for most AUD/SUD treatment settings. While a single-question tool exists (Cuijpers and Smit, 2001), it is designed to assess risk for children of those with AUD/SUD and does not provide additional therapeutic benefit that our tool appears to offer patients. Other similar tools require specialized psychiatric genetics (Costain et al., 2014a, Austin et al., 2014, Austin, 2019) or social work (Mancini, 2021, Samson et al., 2019) training for pedigree or genogram analysis, respectively. In addition to these tools mostly being for research, not clinical, purposes, another limitation is that family history is sometimes a small part of a larger research study and thus investigative teams are forced to prioritize breadth over depth.

However, these tools have provided invaluable information that have propelled the field forward. For example, it is well-documented that evidence-based psychiatric genetic counseling sessions with patients who struggle with mental health disorders have positive psychological and knowledge impacts for both patients and family members (Costain et al., 2014b, Hippman et al., 2016, Inglis et al., 2015, Moldovan et al., 2017, Costain et al., 2014a). Examples of improved domains include disease perception, knowledge of disease etiology, satisfaction with treatment, empowerment, stigma, self-efficacy, and disease symptoms (Costain et al., 2014b, Hippman et al., 2016, Inglis et al., 2015, Moldovan et al., 2017, Costain et al., 2014a). As stated, though, the training takes years, there are few counselors trained in this subfield, and there are few schools that offer such training (Moldovan et al., 2019, Austin, 2019). In contrast, genograms have been widely used in social work for decades to help patients with mental health disorders as well as AUD/SUD (Huss and Kapulnik, 2021), but there are few published scientific inquiries into this practice or its impact on treatment outcomes.

### 1.2 Present study

The purpose of the tool discussed in this study is to offer a way for anyone working at an inpatient or outpatient addiction facility to provide a patient with their family density of AUD/SUD in a simple yet thorough manner. This addresses the aforementioned gaps in providing patients with clinically actionable non-molecular genetic information while providing a valuable research measure. Briefly, the tool provides family density information (similar to Pandey et al., 2020 and Khoddam et al., 2015) via a pedigree assessment. The information from the pedigree is then transferred to an algorithm that provides a numeric density score. The intended use is to provide patients with a new way of visualizing (via the pedigree) and thinking (via the score) about their family history that can positively affect their disease knowledge, psychology, and treatment. Thus, the family history protocol is designed to be therapeutic on two levels – first, conducting the family history protocol is inherently reflective, and second, debriefing about it with a trained specialist or clinician provides an opportunity for intervention. It is designed to provide patients with a sense of the degree to which genetic factors might influence their AUD/SUD and can be an aid in understanding the role of gene-environment interplay.

The aims of this study are as follows. First, to describe the novel protocol for assessing patients’ family density of AUD/SUD. Second, to provide the algorithm for transferring the pedigree to a numerical density value. Third, to present the aggregated results for this density score from pilot testing over two years at an inpatient addiction treatment facility. To clarify, testing the effect of the tool on treatment, knowledge, prevention, or other clinical outcomes was outside the scope of the current study and will be examined later.

## 2 Methods

### 2.1 Sample and approach

Participants included *N* = 871 (*M*age = 40.09 years; Female = 35.71 %; White = 85.10 %; Hispanic or Latino/a = 8.94 %) patients at a short-term inpatient addiction treatment facility. See Table 1 for a full demographic breakdown. The treatment facility is located in the New York City metropolitan area and accepts most major insurances and is in partnership with several local unions but does not accept Medicaid or Medicare. It is a new facility designed to close the research-to-practice gap with science-based care, evidence-based practice, and research. Average length of stay for this subset of the patient population is 22.50 days (range 2–43 days) across both stabilization (detox) and rehabilitation levels of care. Typical referrals are self-referrals and private referrals from local providers and facilities.

**Table 1:** Demographic and clinical indicator breakdown of participant sample.

Age	*M*= 40.09 (SD=13.62) Range 18–82
Sex	
Male	560 (64.29 %)
Female	311 (35.71 %)
Race	
American Indian or Alaska Native	4 (0.51 %)
Asian	12 (1.52 %)
Black	25 (3.16 %)
Native Hawaiian or Other Pacific Islander	2 (0.25 %)
White	674 (85.10 %)
Another	75 (9.47 %)
Did not provide response or Missing	79
Additional Race (*n* = 27)	
American Indian or Alaska Native	0
Asian	4 (14.81 %)
Black	3 (11.11 %)
Native Hawaiian or Other Pacific Islander	0
White	4 (14.81 %)
Another	16 (59.26 %)
Hispanic or Latino/a	
Yes	68 (8.94 %)
No	693 (91.06 %)
Did not provide response or Missing	110
Primary substance	
Alcohol	560 (64.29 %)
Amphetamines	20 (2.30 %)
Cannabis	41 (4.71 %)
Cocaine	53 (6.08 %)
Hallucinogens	7 (0.80 %)
Hypnotics	1 (0.11 %)
Inhaled toxins	5 (0.57 %)
Nicotine	1 (0.11 %)
Opioids	128 (14.70 %)
Sedatives	48 (5.51 %)
Steroids	0
Synthetic cannabinoids	0
Other	7 (0.80 %)
Secondary substance (*n* = 442)	
Alcohol	58 (13.52 %)
Amphetamines	16 (3.73 %)
Cannabis	129 (30.07 %)
Cocaine	96 (22.38 %)
Hallucinogens	3 (0.70 %)
Hypnotics	3 (0.70 %)
Inhaled toxins	1 (0.23 %)
Nicotine	1 (0.23 %)
Opioids	44 (10.26 %)
Sedatives	71 (16.55 %)
Steroids	1 (0.23 %)
Synthetic cannabinoids	0
Other	6 (1.40 %)
Tertiary substance (*n* = 182)	
Alcohol	26 (14.29 %)
Amphetamines	10 (5.49 %)
Cannabis	44 (24.18 %)
Cocaine	36 (19.78 %)
Hallucinogens	10 (5.49 %)
Hypnotics	0
Inhaled toxins	0
Nicotine	2 (1.10 %)
Opioids	19 (10.44 %)
Sedatives	25 (13.73 %)
Steroids	0
Synthetic cannabinoids	0
Other	10 (5.49 %)
AUD/SUD Symptom Severity*	
Primary substance reported	*M*= 8.94 (SD=2.18); range 0–11
Secondary substance reported	*M*= 7.24 (SD=3.73); range 0–11

⁎ Did not screen for tertiary substance severity

All patients at this facility underwent a comprehensive rehabilitation assessment that was conducted by a research specialist wherein information was collected at baseline about their substance use history, abstinence-related cognitions, psychosocial functioning, and social network. This baseline assessment took up to 2 h to complete and was considered treatment as usual, thereby this project was deemed “not human subjects research” (Pearl ID 2023–0158). Following the assessment, a clinical report was written and provided to the patient’s treatment team by the research specialists’ supervisor who was the director of the research department (Bourdon et al., 2024). Patients had the option to review the report with their primary clinician or the lead researcher (JLB), with a minority of patients choosing to do so (the number who chose to review their results was not tracked). Assessment data were collected from February 2021 through February 2023.

### 2.2 Measure and variables of interest

The key measure and variables of interest pertain to the latter section of the assessment, social network. Within that section is a novel measure to screen for Family Density of AUD/SUD. Per the aims of this paper, the main description of the protocol and algorithm for getting the final Family Density Score will be in the “Results” section below, along with participants’ aggregated scores. Variables of interest include the final Family Density Score and key demographics of interest (sex, race, ethnicity, sexual orientation, primary substance).

### 2.3 Analyses

Analyses for all variables of interest include descriptives (mean, standard deviation, standard error, skew, kurtosis) as well as means difference tests to examine Family Density Score differences among key demographic variables (sex [female vs. male], race [White vs. non-White], ethnicity [Hispanic vs. non-Hispanic], primary substance [alcohol vs. non-alcohol], sexual orientation [heterosexual vs. not heterosexual], age). Specifically, t-tests were for independent variables with two groups and correlation for continuous variables (i.e., age).

## 3 Results

### 3.1 Protocol (aim 1)

The current protocol was based on the Family Informant Schedule and Criteria (FISC; used in Stoltenberg et al., 1998 and originated in Mannuzza et al., 1985) and the Family Expression of Alcohol (FEA; Zucker et al., 1994; Stoltenberg et al., 1998). See Appendix A for the exact protocol script.

**Fig. 1:**
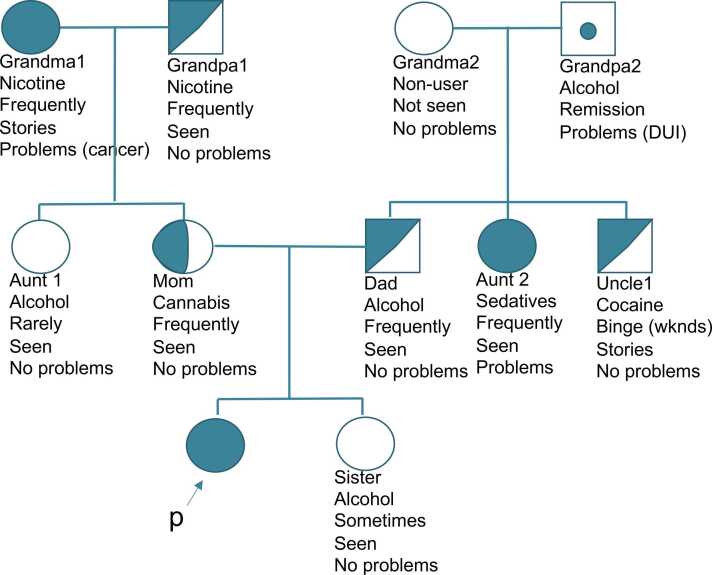
Example family pedigree per the Family Density protocol.

The protocol involved asking patients key questions about their 1st degree (50 % genetic relatedness) and 2nd degree (25 % genetic relatedness) relatives’ alcohol and substance use patterns regardless of diagnosed disorder. This was done interactively with the patient by creating a pedigree one family member at a time and marking key information below that family member’s pedigree symbol (see Fig. 1 as an example). Squares were used for men, circles for women, and diamonds for non-binary, genderfluid, or genderqueer relatives.

Four key questions were asked of each relative: (1) *What substance does/did your relative use the most or have problems with?* (2) *How often do/did they use?* (3) *Can you confirm that you saw this person using or that you remember hearing stories of their use from trusted relatives?* (4) *Did your relative ever have problems or consequences from using this substance? Examples include legal, marital / relationship, work, health, fights, attending treatment, financial, etc.* See Table 2 for the full questions and answer options. Depending on how patients answered, family members pedigree shapes were fully shaded (definite use disorder), half shaded (probable use disorder), or left unshaded. A dot was used for those in recovery, but they functioned the same as a fully shaded person in the algorithm.

**Table 2:** Pedigree questions and answer options.

Question	Answer Options	Shading
What substance does/did your relative use the most or have problems with?	1. Alcohol 2. Amphetamines 3. Cannabis 4. Hallucinogens 5. Hypnotics 6. Inhaled toxins 7. Opioids 8. Sedatives 9. Steroids 10. Synthetic cannabinoids 11. Other 12. None	• Full shading: Options 1–11 • Half shading: Options 1–11 • Dot: Options 1–11 • No shading: Option 12
How often do/did they use?	1. Non-user (never used any substance) 2. In recovery or abstinent (used to use but does not anymore) 3. Rarely 4. On special occasions / sometimes 5. Frequently and/or binges	• Full shading: Option 5 • Half shading: Option 5 • Dot: Option 2 • No shading: Options 1, 3, 4
Can you confirm that you saw this person using or that you remember hearing stories of their use from trusted relatives?	1. Yes 2. No	• Full shading: Option 1 • Half shading: Option 1 • Dot: Option 1 • No shading: Option 2
Did your relative ever have problems or consequences from using this substance? Examples include legal, marital / relationship, work, health, fights, attending treatment, financial, etc.	1. Yes 2. No	• Full shading: Option 1 • Half shading: Option 2 • Dot: Option 1 • No shading: Option 2

The protocol allowed those who were adopted to create a pedigree for their adoptive family (a measure of environment that was visually useful to patients and which had a resulting score of “0” for the Family Density Score), natal family (if known), or both. Half-relatives could also be accounted for, but any relative with a relatedness of 12.50 % or less was ultimately dropped from the final score calculation.

It is paramount to note that while the pedigree was captured via pen and paper, the script was followed electronically via REDCap (Harris et al., 2009). Additionally, the paper pedigree was transferred into REDCap after the assessment to allow the data to be downloaded and for the Density Score to be calculated (see below) and added to a clinical assessment report. This entire process was done by a research specialist as part of a larger assessment procedure (see *Sample and Approach*). The completion of the report (see Bourdon et al., 2024) was then communicated to the treatment team by the research specialist’s supervisor and director of the research department.

### 3.2 Algorithm (aim 2)

The current algorithm is based on Pandey’s (2020) work to form a family history measure of AUD that took density into account, which is a more sophisticated version of the FEA (FEA; Zucker et al., 1994; Stoltenberg et al., 1998). See Appendix B for the exact algorithm scoring guidelines and Appendix C for the exact algorithm script in R (R Core Team, 2023). Following the assessment, the assessment specialist calculated the score as follows: (1) Downloaded the electronic pedigree information from REDCap and uploaded data into R. (2) Calculated affected status for all possible relatives. (3) Created a relatedness variable and sum score. (4) Performed the calculations to get the final Family Density Score. The algorithm for the Score is A*W / sum(W) where A =  affectedness of each relative, W =  weight of each category of relative, sum(W) =  the genetic relatedness sum, and A*W =  the sum score of these two data frames using the dot product (multiplication method used with two vectors of equal length to get a single number).

### 3.3 Family density score descriptives (aim 3)

The mean Family Density Score across all patients was 0.47 (standard deviation [SD]=0.24; standard error [SE]=0.01; range=0–1) with a normal distribution (skew=0.06; kurtosis=-0.64). There were significant differences such that females had higher scores than males (*t* = 2.96; df=594.80; *p* = 0.003), White patients had higher scores than non-White patients (*t* = 2.88; *df*=124.74; *p* = 0.005), and those whose primary substance was alcohol had higher scores than those with non-alcohol as a primary substance (*t* = 2.75; *df*=570.00; *p* = 0.006) (see Table 3). Age (*r* = 0.06, *p* = 0.106) and ethnicity (*t* = -.055; df=69.75; *p* = 0.582) were unrelated to Family Density, although there were likely too few in the latter groups to draw conclusions.

**Table 3:** Full results from assessing differences in the Family Density Score with key demographic variables.

Demographic / Independent Variable	Test Performed	Results
Sex	*t-*test (female vs. male)	Female: *M*= 0.50; SD= 0.23 Male: *M*= 0.45; SD= 0.24 *t* = 2.96; df= 594.80; *p* = 0.003 difference= 0.05; CI= 0.02–0.09
Race	*t-*test (White vs. not White)	White: *M*= 0.48; SD= 0.23 Not White: *M*= 0.41; SD= 0.24 *t* = 2.88; df= 124.74; *p* = 0.005 difference= 0.07; CI= 0.02–0.13
Primary substance	*t-*test (alcohol vs. non-alcohol)	Alcohol: *M*= 0.49; SD= 0.24 Non-alcohol: *M*= 0.44; SD= 0.23 *t* = 2.75; df= 570.00; *p* = 0.006 difference= 0.05; CI= 0.01–0.08
Ethnicity	*t-*test (Hispanic or Latino/a vs. not Hispanic or Latino/a)	Hispanic or Latino/a: *M*= 0.45; SD= 0.25 Not Hispanic or Latino/a: *M*= 0.47; SD= 0.23 *t* = −0.55; df= 69.75; *p* = 0.582 difference= 0.02; CI= −0.08–0.05
Sexual orientation	*t-*test (heterosexual vs. not heterosexual)	Heterosexual: *M*= 0.47; SD= 0.23 Not heterosexual: *M*= 0.51; SD= 0.25 *t* = −1.43; df= 103.91; *p* = 0.154 difference= 0.04; CI= −0.10–0.02
Age	Pearson correlation	r = 0.58; *p* = 0.106; CI= −0.01–0.13

Notes: SD =  standard deviation; CI =  confidence interval

The mean number of family members affected (probable or definite use disorder) by significant substance use was 5.29 (SD=3.17; range=0–21). Specifically, 52 % of patients reported at least one affected parent, 47 % reported at least one affected sibling, 44 % reported at least one affected avuncular (aunt or uncle), and 38 % reported at least one affected grandparent. Affected avuncular were driven by uncles; 54 % of patients reported at least one affected uncle while 37 % reported at least one affected aunt.

## 4 Discussion

The purpose of the current paper was to provide the protocol (see Results and Appendix A), scoring (see Results and Appendix B), and algorithm (see Results and Appendix C) for conducting and analyzing a new family history of AUD/SUD protocol. Additionally, we offered descriptive results of the Family Density Score from a pilot test of the protocol at an inpatient addiction treatment facility. To our knowledge, no other protocol like this exists and it is the first of its kind designed with the intention to be used by an array of clinicians outside of the psychiatric genetic counseling field (Costain et al., 2014a, Austin et al., 2014, Austin, 2019). The protocol does require training but overall it is simple to use, easy to understand, and aligns with current clinical practice focused on familial risk for specific diseases (e.g., GRACE, Arar et al., 2013; MeTree, Voils et al., 2023). The current study differs from past studies in that it focuses on the utilization of the family history protocol instead of its association with AUD/SUD and related outcomes. This is to help progress in the utilization of family history information toward a clinically-actionable area of research and practice.

### 4.1 The family history protocol

A key study that informed this protocol examined dichotomous family history versus a family density score and found that density offered greater strength of association with outcomes, indicating that it is an overall better measure for research (Pandey et al., 2020). In other words, while dichotomous family history (e.g., presence or absence of familial AUD/SUD) and density (e.g., taking relatedness into account) both predict addiction-related outcomes, the latter offers a more nuanced presentation (Pandey et al., 2020). Thus, the current study expands this work by offering a trainable, useable protocol that can be implemented across behavioral health/psychiatric fields. To date, most examinations of clinical family history protocols have involved diabetes, cardiovascular disease, and/or cancer (Brook et al., 2023; Goldstein et al., 2019; Orlando et al., 2011; Ramsey et al., 2005; J. Rodriguez, 2016; V. Rodríguez et al., 2016; Szczerbiński et al., 2019; Voils et al., 2023; Vornanen et al., 2016). By focusing on AUD/SUD, the current protocol pushes the study of clinical application of family history forward. AUD/SUD is a highly stigmatized disorder that impacts the whole family of the affected person (U.S. Health and Human Services HHS, 2016). Such a tool has vast possibilities for expansion; it may assist with communication about the disorder, knowledge about AUD/SUD, lead to behavior change, help clinicians with case conceptualization, and much more (see *Clinical Implications*). Although the protocol could easily be applied to non-psychiatric/behavioral disciplines, additional research should verify this.

### 4.2 Pilot data aggregated results

The aggregated results of the Family Density Score provide potential insights that must be investigated in future studies across samples. In the current sample, the mean Density Score was 0.47, mirroring the ~0.50 population-level heritability for AUD/SUD (Kendler et al., 2003). Heritability means that across the population of those affected with AUD/SUD, roughly 50 % of the variance of the trait is due to genetic factors (Kendler et al., 2003). Similarly, across our sample the average person’s familial liability for AUD/SUD was 47 % as presented by the Family Density Score. While not a breakdown of genetic variance, the Family Density Score is intended to guide patients and clinicians as to the approximate familial liability for AUD/SUD (i.e., possibly a proxy for genetic liability). It is not meant to be interpreted in a deterministic manner, nor does it encompass environmental risks that can accompany familial AUD/SUD.

Further, the average number of affected family members was five, and this is the first study that we are aware of to report the average number of affected family members, although Andreasen and colleagues noted the lifetime prevalence for alcohol and drug use disorder separately (1986). While not weighted by family size, such a number is still important as it can tell clinicians at a similar treatment facility that, on average, their patients will have five affected family members. Of those five family members, they are more likely to be, in order, uncles, parents, siblings, grandparents, and aunts. We acknowledge that this average is specific to our location and client population and may not generalize in future studies. We also note that family history historically underestimates affected family members when compared to a family study method where each family member is interviewed (Andreasen et al., 1977, Andreasen et al., 1986). Such underestimation is relatively low for AUD/SUD, though, and AUD/SUD are stigmatized diseases that family members may deny having (Andreasen et al., 1986). When taken together, understanding the Density Score and who is likely to be affected in an average patient has clinical implications for treatment, family therapy, education, and addressing stigma.

Additionally, there were sex, race, and primary substance differences that again, remain to be replicated in different settings. Female patients (compared to males), White patients (compared to all other races), and patients whose primary substance was alcohol (compared to all other substances) all had higher Family Density Scores. The sex and gender differences align with Pandey et al. (2020), but there is no comparable literature for the difference in primary substance. Regarding race, it is known that there are racial disparities in rates of AUD/SUD with many possible and intertwined explanations, and White individuals are more likely to receive treatment for AUD (Vaeth et al., 2017, Zemore et al., 2019). However, there is little information about racial differences relative to family history. This needs to be further explored, and culturally competent protocols should be developed in the future.

Females are less likely to be diagnosed with AUD and to seek treatment (Salvatore et al., 2017, Zemore et al., 2019). The current findings add more evidence that it is critical to reduce barriers to access for females given that they had higher Family Density Scores than males. There are many possible reasons for the current pattern, including that greater family density of AUD/SUD may be a motivating factor for females to seek treatment given the complex barriers they otherwise face compared to males (Pinedo et al., 2019).

### 4.3 Clinical implications

We hope that this tool can be utilized across treatment settings (outpatient, inpatient, intensive outpatient, medical) and providers (social worker, psychologist, psychiatrist, mental health counselor, medical provider). While clinical utility and behavior change were not robustly studied in this pilot phase, nor do we offer concrete guidance on how to use this tool, we can offer some insight based on our experiences creating this protocol. In regard to guidance and clinical utility, the opportunity was offered to every patient to meet with their primary clinician or the lead researcher (author JLB) to review the results of the full assessment, including their pedigree. Dozens of patients opted into this, usually opting to meet with the lead researcher due to constraints on the clinical team. During this time their Score was presented to them, creating a natural chance to discuss perceived accuracy, emotions about seeing the Score, and how to navigate what the Score means. This presents the foundation for future guidance and utility, and we plan to combine this with current psychiatric genetic protocols on how to discuss family history (Austin, 2019). In regard to behavior change, patients often shared that they found the experience therapeutic and the ability to visually and numerically think about their family history as insightful. They often asked questions about the risk posed to their children or asked questions about how to discuss this with their family members.

From a clinical perspective, it may help providers identify patients who are at risk for specific types of AUD/SUD and/or comorbidities (Schepis et al., 2023) and adjust treatment and prevention efforts accordingly. In addition to benefiting patients via improving education, attitudes, and disease knowledge (Costain et al., 2014a, Austin et al., 2014, Austin, 2019), this tool also holds promise for assisting unaffected family members. For example, the current Family Density protocol could be utilized in family counseling to help children of patients understand their risk for AUD/SUD and open the lines of family communication, which is important given that the stigmatization of SUDs extends to patients' family members (O'Shay-Wallace, 2020). This has been shown to improve family cohesion and flexibility in protocols for cancer (V. Rodríguez et al., 2016), though, given that use disorders are stigmatized differently than other health conditions these conversations would be navigated best by family therapists. It may also affect the perceived harm, sense of self-control, motivation, behavior change, and other such factors among family members and patients alike, as it goes beyond perceived risk that past studies have examined (Dingel et al., 2019, Vornanen et al., 2016) by providing a concrete Family Density Score. Finally, assessing family history is a key part of targeted AUD/SUD prevention strategies across multiple age groups (Afuseh et al., 2020). This Family Density protocol can help providers share more individualized information about a person’s risks for developing disorder, thus empowering them to make informed decisions about their future alcohol or drug use.

### 4.4 Limitations

The current study has limitations to note. First, we deemed the influence of anyone related less than 2nd degree to be minimal. The protocol allows for third-degree and non-genetically related family members to be captured, but they do not count in the algorithm. Second, the scoring provided in Appendix C is written for R (R Core Team, 2023) and has not been adopted into other coding languages, which may limit accessibility for clinicians who are unlikely to be familiar with R. The algorithm could be transferred into other platforms relatively easily, and interested researchers and clinicians can reach out to the primary author for help doing this. Third, additional information such as length of time lived with a family member, whether family members were still alive, and in-law relatives were initially captured in the pedigree to have a complementary Environmental Density Score. Such additional information quickly proved too cumbersome for patients, made the protocol take over 30 minutes, and was thus dropped. Future studies could resume a separate study of such information. Fourth, the demographics of the patients in this study are not representative of the wider US and thus the descriptives of the Family Density Score may not generalize to other facilities and/or geographic areas. Finally, the psychometrics of the Family Density Score were not addressed in this study. We plan to assess the reliability and validity of the Family Density protocol in future studies. However, this does not undercut its therapeutic and clinical utility.

### 4.5 Future directions and conclusions

Future studies are needed to examine the clinical impact of the protocol, ideally using a randomized clinical trial design. There is ample evidence to suggest that such a novel protocol would have an impact on patients’ knowledge, disease conceptualization, family communication, etiology, and other related components of their AUD/SUD. However, such examinations were outside the scope of the present study. The intended use of the protocol is to impact care in a positive way by providing clinicians with an easy-to-use tool that incorporates genetic information (via family history) and to provide patients with a new way of thinking about their family history. The long-term goal is to be able to incorporate this Family Density Score into standard family therapy across treatment centers for AUD/SUD. Thus, we hope that this work can be done across research and clinical settings by making the protocol freely available. This pilot test was overall successful, providing aggregated information about the average family history of patients at an inpatient addiction treatment facility while taking steps to close the research-to-practice gaps in the field.

## Funding source

This research did not receive any specific grant from funding agencies in the public, commercial, or not-for-profit sectors.

## Author contributions

JLB conceived of the project and initiated the data collection protocols. JW conducted a literature search. JB and JW wrote the initial drafts of the paper while SV provided edits and formatting to early drafts. MWF and VM were consulted and provided edits on various paper drafts. All authors edited the final draft of the paper.

## Statement on informed consent

Informed consent was not needed because this study is a secondary analysis of deidentified data that were initially collected for quality and treatment purposes. It does not constitute human subjects research (Pearl ID 2023–0158).

## CRediT authorship contribution statement

**Verdecanna Sabrina:** Writing – review & editing, Resources. **Wright Jordan:** Writing – original draft, Resources. **Bourdon Jessica Lynn:** Writing – review & editing, Writing – original draft, Visualization, Validation, Supervision, Software, Resources, Project administration, Methodology, Investigation, Formal analysis, Data curation, Conceptualization. **McCutcheon Vivia V.:** Writing – review & editing, Supervision, Conceptualization. **Francis Mer W.:** Writing – review & editing, Conceptualization.

## Declaration of Competing Interest

The authors declare that they have no known competing financial interests or personal relationships that could have appeared to influence the work reported in this paper.

## Footnotes

Appendix Supplementary data associated with this article can be found in the online version at doi:10.1016/j.dadr.2025.100321.
