# Supplementary material for: Protocol for a new family history of addiction density score to aid in the treatment of alcohol and substance use disorders
Source: Drug Alcohol Depend Rep. 2025 Feb 20;15:100321. doi: 10.1016/j.dadr.2025.100321 (PMC11930440; doi:10.1016/j.dadr.2025.100321)
Supplement: Supplementary file 1 — Supplementary material [file mmc1.docx]

**APPENDIX A: PROCEDURES / SCRIPT**

**PROCEDURES / SCRIPT**

**Script:** This next section concerns your family history of substance use disorder. I’m going to ask you a series of questions. Please answer to the best of your ability.

I want to know the family density of substance use in your family. I’m going to ask you to think back about whether your family members used a substance, even occasionally, and which substance they used the most. For most people, it will be alcohol or nicotine, but it could be any substance.

I’m not looking only for problematic substance use, but any and all substance use.

We’re going to focus on 1^st^ degree and 2^nd^ degree relatives. You’re 50% related to 1^st^ degree relatives and 25% related to 2^nd^ degree relatives. 1^st^ degree relatives are biological siblings and parents. 2^nd^ degree relatives are aunts, uncles, and grandparents.

I will use shapes to represent you and your family members. I will use squares for men, circles for women, and diamonds for non-binary family members. I can also represent transgender family members.

Before we begin, are there any non-binary or transgender family members? [if so, then note that, if not then no reason to continually ask sex/gender for family members during the assessment]

Horizontal lines indicate mating and/or marriage, vertical lines indicate birth, dashed are adoption, and diagonal lines indicate that someone has passed away.

I use solid lines for biological relatedness and dashed lines if anyone is adopted. Please let me know if you or anyone in your family is adopted as we go.

Importantly, a fully shaded shape indicates that someone likely has a substance use disorder, half shaded indicates they may have it, and a dot indicates that they are in recovery or remission.

All of this goes into an algorithm that will give a standardized number between 0 and 1. That number will go onto your report that I will give your primary therapist.

Let’s begin. This is you with an arrow pointing toward you. I have shaded your [CIRCLE / SQUARE / DIAMOND] in because you have a substance use disorder and written “P” for proband, a genetic counseling term. For our purposes, it means “patient.”

First, were you adopted?

- IF YES, then instructions pop up to put 0 for all relatedness questions

Do you have any siblings?

- Are they FULL, HALF, ADOPTED?
- To your knowledge, what substance did RELATIVE use the most or have problems with?
- How often did they use SUBSTANCE? Did they have a chronic binge pattern of use?
- ANSWER OPTIONS:
  - - - None b/c remission / recovery (non-user)
      - None b/c never used in lifetime (non-user)
      - Rarely
      - Sometimes / on special occasions
      - Frequently OR binged
- Can you confirm that you did see this person using OR that there are stories you heard from family members such that you know for a fact they used SUBSTANCE? [if patient is unsure, clarify that this is a validity check - we have to be sure the person did use the substance]
- Did relative ever have problems / consequences from using SUBSTANCE? Problems are somewhat subjective to your family and your perspective, but examples include: legal, marital / relationship, work, health, fights, in treatment, financial, etc.

**[CONTINUE ONTO PARENTS, AUNTS, UNCLES, GRANDPARENTS]**

**NOTES FOR ASSESSOR:**

- Construct a pedigree around the patient of 1^st^ and 2^nd^ degree relatives
  - Pedigree will begin with the proband’s current generation (siblings) then move out to parents, grandparents, aunts, uncles
  - Be sure to ask carefully and go slowly - make sure that family members aren’t omitted due to death, estrangement, etc.
  - Include ALL family members, even if they do not have a history of substance use and even if they are deceased
  - Do not include family members who are only part of the family via marriage EXCEPT for step-relatives (step parents, step grandparents)
  - Be sure to confirm along the way any step parents, step siblings, half siblings, half-relations via multiple marriages, etc.
- For each relative, as you go, ask the above questions
- Be sure to review it and make sure that it’s clear to the patient
- If necessary, affirm that many patients enjoy seeing the pedigree
- Reminder:
  - DEFINITE (full shade) = yes to all questions; there are noted problems / consequences from relatives’ use (includes remission if problems also checked)
  - PROBABLE (half shade) = yes to all questions but problems / consequences
  - REMISSION / RECOVERY (dot) = used to use heavily but stopped (falls into probable if no problems endorsed)

- Encourage patients to do their best guess if they do not know – after all, perception of others’ use is powerful and more relevant than others’ actual use (social norms theory)
- Follow notes on how to construct pedigree
- Be mindful and respectful of transgender, gender non-conforming, intersex, or other non-binary gender identities and sexes of the patient and/or their family members
- Symbols for non-binary genders and sexes will be provided
  - **While pedigrees reflect SEX, transgender and gender non-conforming individuals may be on hormones or have undergone transition therapy to make their sex align with their gender. Therefore, the symbols used are inferring both sex and gender with transgender, non-binary, gender non-conforming, genderqueer, and other identities being properly indicated
- Initially refer to family members as “they” or “this relative” or “this person” instead of assuming “he” and “she”
- Include step and adopted relatives in the pedigree

**REMINDERS FOR ASSESSOR:**

- **Symbols:**
  - Cisgender man: square
  - Cisgender woman: circle
  - Transgender man: square with “AFAB” written underneath (assigned female at birth)
  - Transgender woman: circle with “AMAB” written underneath (assigned female at birth)
  - Gender non-conforming, non-binary, genderqueer, another: diamond with “AFAB” or “AMAB” written underneath (ask clarifying language)
  - Horizontal line: mating / married
  - Horizontal line with dash through it: divorced
  - Vertical line: offspring
  - Dotted vertical line: adopted offspring
  - Diagonal line through circle, square, or diamond: deceased
  - Shaded in circle, square, or diamond: affected (for our study, “definite disorder”)
  - Half shaded circle, square, or diamond: probable affected (not typical use of this symbol)
  - Dot in circle, square, or diamond: abstinent (not typical use of this symbol)
  - Dot in circle, square, or diamond: abstinent (not typical use of this symbol)
  - Empty circle, square, or diamond: low-risk (social) user or non-user
  - Arrow with a “P:” proband (i.e., the patient)
  - Can put name underneath the symbol
- **Included family members:**
  - Everyone in the current generation and above, 1^st^ and 2^nd^ degree relatives
  - Do **not** include any offspring of the current generational line (i.e., children, nieces, nephews)
- **Proband:** The patient
- **Family relatedness:**
  - 1^st^ degree relatives:
  - Share 50% of DNA with proband
  - Full siblings
  - Biological parents
  - 2^nd^ degree relatives:
  - Share 25% of DNA with proband
  - Half siblings
  - Biological grandparents
  - Avuncular (aunts, uncles)
  - 0% genetics:
  - If proband was adopted
  - If a family member was adopted
  - Family member by marriage (ex - aunts, uncles, step parents)
- **Name:** While this is not always part of a pedigree, for clinical transparency, it would be useful to include the name of family members under their symbol

**APPENDIX B: SCORING GUIDELINES**

**SCORING**

1. **Affected Status**
   1. Score each person as 0-1 with abstinent, probable, and definite as “1” and non-drinker and social as “0” (making a clear distinction between affected and non-affected)
2. **Relatedness**
   1. Score each person as 0.25 (second degree relative), 0.5 (first degree relative), or 1.0 (monozygotic twin)
3. **Family Density Score**
   1. FHDr = A*W / sum(W)
      1. A = affectedness of each relative; is 0 or 1
      2. W = weighting of each category of relative; W = 1 / 2^D
         1. D = 1 for 1^st^ degree and 2 for 2^nd^ degree
         2. W = 0.5 for 1^st^ degree and 0.25 for 2^nd^ degree
      3. A*W is the sum score for family history density; use dot product to calculate; they are each vectors
      4. A1W1 + A2W2 + A3W3 + … AnWn
      5. Gives us a scalar number 0-1 from two vectors

**APPENDIX C: ALGORITHM FOR CALCULATING FAMILY DENSITY SCORE FOR AN INDIVIDUAL PATIENT**

###

# Load and check data

###

dat <- read.csv("EndpointComprehensiv_DATA_2023-11-09_1533.csv", na.strings = c("", " ", "na", "n/a", "NA", "N/A"), header = TRUE) # !~!~!~!~!~!~!~!~!~!~ # Change file name to the correct file name

head(dat[,1:10]) # too much data to get preview of all of it; just pick a couple of columns

tail(dat[,1:10])

names(dat)

dim(dat)

###

# Pull the data ONLY FOR THE PARTICIPANT OF INTEREST

###

dat2 <- dat[dat$record_id == "13774",] # !~!~!~!~!~!~!~!~!~!~ # Change to the correct participant ID

head(dat2[,1:10]) # too much data to get preview of all of it; just pick a couple of columns

names(dat2)

dim(dat2)

# Created affected status for all possible relatives

fhx_a_sib1_aff <- NULL

fhx_a_sib1_aff[dat2$fhx_a_sib1j == 1 | dat2$fhx_a_sib1j == 2 | dat2$fhx_a_sib1j == 3 | dat2$fhx_a_sib1j == 4] <- 0 # non-user, abstinent, social user

fhx_a_sib1_aff[(dat2$fhx_a_sib1j == 5 | dat2$fhx_a_sib1j == 2) & dat2$fhx_a_sib1k == 1 & dat2$fhx_a_sib1l == 0] <- 1 # probable

fhx_a_sib1_aff[(dat2$fhx_a_sib1j == 5 | dat2$fhx_a_sib1j == 2) & dat2$fhx_a_sib1k == 1 & dat2$fhx_a_sib1l == 1] <- 1 # definite

fhx_a_sib1_aff

fhx_a_sib2_aff <- NULL

fhx_a_sib2_aff[dat2$fhx_a_sib2j == 1 | dat2$fhx_a_sib2j == 2 | dat2$fhx_a_sib2j == 3 | dat2$fhx_a_sib2j == 4] <- 0 # non-user, abstinent, social user

fhx_a_sib2_aff[(dat2$fhx_a_sib2j == 5 | dat2$fhx_a_sib2j == 2) & dat2$fhx_a_sib2k == 1 & dat2$fhx_a_sib2l == 0] <- 1 # probable

fhx_a_sib2_aff[(dat2$fhx_a_sib2j == 5 | dat2$fhx_a_sib2j == 2) & dat2$fhx_a_sib2k == 1 & dat2$fhx_a_sib2l == 1] <- 1 # definite

fhx_a_sib2_aff

fhx_a_sib3_aff <- NULL

fhx_a_sib3_aff[dat2$fhx_a_sib3j == 1 | dat2$fhx_a_sib3j == 2 | dat2$fhx_a_sib3j == 3 | dat2$fhx_a_sib3j == 4] <- 0 # non-user, abstinent, social user

fhx_a_sib3_aff[(dat2$fhx_a_sib3j == 5 | dat2$fhx_a_sib3j == 2) & dat2$fhx_a_sib3k == 1 & dat2$fhx_a_sib3l == 0] <- 1 # probable

fhx_a_sib3_aff[(dat2$fhx_a_sib3j == 5 | dat2$fhx_a_sib3j == 2) & dat2$fhx_a_sib3k == 1 & dat2$fhx_a_sib3l == 1] <- 1 # definite

fhx_a_sib3_aff

fhx_a_sib4_aff <- NULL

fhx_a_sib4_aff[dat2$fhx_a_sib4j == 1 | dat2$fhx_a_sib4j == 2 | dat2$fhx_a_sib4j == 3 | dat2$fhx_a_sib4j == 4] <- 0 # non-user, abstinent, social user

fhx_a_sib4_aff[(dat2$fhx_a_sib4j == 5 | dat2$fhx_a_sib4j == 2) & dat2$fhx_a_sib4k == 1 & dat2$fhx_a_sib4l == 0] <- 1 # probable

fhx_a_sib4_aff[(dat2$fhx_a_sib4j == 5 | dat2$fhx_a_sib4j == 2) & dat2$fhx_a_sib4k == 1 & dat2$fhx_a_sib4l == 1] <- 1 # definite

fhx_a_sib4_aff

fhx_a_sib5_aff <- NULL

fhx_a_sib5_aff[dat2$fhx_a_sib5j == 1 | dat2$fhx_a_sib5j == 2 | dat2$fhx_a_sib5j == 3 | dat2$fhx_a_sib5j == 4] <- 0 # non-user, abstinent, social user

fhx_a_sib5_aff[(dat2$fhx_a_sib5j == 5 | dat2$fhx_a_sib5j == 2) & dat2$fhx_a_sib5k == 1 & dat2$fhx_a_sib5l == 0] <- 1 # probable

fhx_a_sib5_aff[(dat2$fhx_a_sib5j == 5 | dat2$fhx_a_sib5j == 2) & dat2$fhx_a_sib5k == 1 & dat2$fhx_a_sib5l == 1] <- 1 # definite

fhx_a_sib5_aff

fhx_a_sib6_aff <- NULL

fhx_a_sib6_aff[dat2$fhx_a_sib6j == 1 | dat2$fhx_a_sib6j == 2 | dat2$fhx_a_sib6j == 3 | dat2$fhx_a_sib6j == 4] <- 0 # non-user, abstinent, social user

fhx_a_sib6_aff[(dat2$fhx_a_sib6j == 5 | dat2$fhx_a_sib6j == 2) & dat2$fhx_a_sib6k == 1 & dat2$fhx_a_sib6l == 0] <- 1 # probable

fhx_a_sib6_aff[(dat2$fhx_a_sib6j == 5 | dat2$fhx_a_sib6j == 2) & dat2$fhx_a_sib6k == 1 & dat2$fhx_a_sib6l == 1] <- 1 # definite

fhx_a_sib6_aff

fhx_a_sib7_aff <- NULL

fhx_a_sib7_aff[dat2$fhx_a_sib7j == 1 | dat2$fhx_a_sib7j == 2 | dat2$fhx_a_sib7j == 3 | dat2$fhx_a_sib7j == 4] <- 0 # non-user, abstinent, social user

fhx_a_sib7_aff[(dat2$fhx_a_sib7j == 5 | dat2$fhx_a_sib7j == 2) & dat2$fhx_a_sib7k == 1 & dat2$fhx_a_sib7l == 0] <- 1 # probable

fhx_a_sib7_aff[(dat2$fhx_a_sib7j == 5 | dat2$fhx_a_sib7j == 2) & dat2$fhx_a_sib7k == 1 & dat2$fhx_a_sib7l == 1] <- 1 # definite

fhx_a_sib7_aff

fhx_a_sib8_aff <- NULL

fhx_a_sib8_aff[dat2$fhx_a_sib8j == 1 | dat2$fhx_a_sib8j == 2 | dat2$fhx_a_sib8j == 3 | dat2$fhx_a_sib8j == 4] <- 0 # non-user, abstinent, social user

fhx_a_sib8_aff[(dat2$fhx_a_sib8j == 5 | dat2$fhx_a_sib8j == 2) & dat2$fhx_a_sib8k == 1 & dat2$fhx_a_sib8l == 0] <- 1 # probable

fhx_a_sib8_aff[(dat2$fhx_a_sib8j == 5 | dat2$fhx_a_sib8j == 2) & dat2$fhx_a_sib8k == 1 & dat2$fhx_a_sib8l == 1] <- 1 # definite

fhx_a_sib8_aff

fhx_a_sib9_aff <- NULL

fhx_a_sib9_aff[dat2$fhx_a_sib9j == 1 | dat2$fhx_a_sib9j == 2 | dat2$fhx_a_sib9j == 3 | dat2$fhx_a_sib9j == 4] <- 0 # non-user, abstinent, social user

fhx_a_sib9_aff[(dat2$fhx_a_sib9j == 5 | dat2$fhx_a_sib9j == 2) & dat2$fhx_a_sib9k == 1 & dat2$fhx_a_sib9l == 0] <- 1 # probable

fhx_a_sib9_aff[(dat2$fhx_a_sib9j == 5 | dat2$fhx_a_sib9j == 2) & dat2$fhx_a_sib9k == 1 & dat2$fhx_a_sib9l == 1] <- 1 # definite

fhx_a_sib9_aff

fhx_a_sib10_aff <- NULL

fhx_a_sib10_aff[dat2$fhx_a_sib10j == 1 | dat2$fhx_a_sib10j == 2 | dat2$fhx_a_sib10j == 3 | dat2$fhx_a_sib10j == 4] <- 0 # non-user, abstinent, social user

fhx_a_sib10_aff[(dat2$fhx_a_sib10j == 5 | dat2$fhx_a_sib10j == 2) & dat2$fhx_a_sib10k == 1 & dat2$fhx_a_sib10l == 0] <- 1 # probable

fhx_a_sib10_aff[(dat2$fhx_a_sib10j == 5 | dat2$fhx_a_sib10j == 2) & dat2$fhx_a_sib10k == 1 & dat2$fhx_a_sib10l == 1] <- 1 # definite

fhx_a_sib10_aff

fhx_a_parent1_aff <- NULL

fhx_a_parent1_aff[dat2$fhx_a_parent1j == 1 | dat2$fhx_a_parent1j == 2 | dat2$fhx_a_parent1j == 3 | dat2$fhx_a_parent1j == 4] <- 0 # non-user, abstinent, social user

fhx_a_parent1_aff[(dat2$fhx_a_parent1j == 5 | dat2$fhx_a_parent1j == 2) & dat2$fhx_a_parent1k == 1 & dat2$fhx_a_parent1l == 0] <- 1 # probable

fhx_a_parent1_aff[(dat2$fhx_a_parent1j == 5 | dat2$fhx_a_parent1j == 2) & dat2$fhx_a_parent1k == 1 & dat2$fhx_a_parent1l == 1] <- 1 # definite

fhx_a_parent1_aff

fhx_a_parent2_aff <- NULL

fhx_a_parent2_aff[dat2$fhx_a_parent2j == 1 | dat2$fhx_a_parent2j == 2 | dat2$fhx_a_parent2j == 3 | dat2$fhx_a_parent2j == 4] <- 0 # non-user, abstinent, social user

fhx_a_parent2_aff[(dat2$fhx_a_parent2j == 5 | dat2$fhx_a_parent2j == 2) & dat2$fhx_a_parent2k == 1 & dat2$fhx_a_parent2l == 0] <- 1 # probable

fhx_a_parent2_aff[(dat2$fhx_a_parent2j == 5 | dat2$fhx_a_parent2j == 2) & dat2$fhx_a_parent2k == 1 & dat2$fhx_a_parent2l == 1] <- 1 # definite

fhx_a_parent2_aff

fhx_a_parent3_aff <- NULL

fhx_a_parent3_aff[dat2$fhx_a_parent3j == 1 | dat2$fhx_a_parent3j == 2 | dat2$fhx_a_parent3j == 3 | dat2$fhx_a_parent3j == 4] <- 0 # non-user, abstinent, social user

fhx_a_parent3_aff[(dat2$fhx_a_parent3j == 5 | dat2$fhx_a_parent3j == 2) & dat2$fhx_a_parent3k == 1 & dat2$fhx_a_parent3l == 0] <- 1 # probable

fhx_a_parent3_aff[(dat2$fhx_a_parent3j == 5 | dat2$fhx_a_parent3j == 2) & dat2$fhx_a_parent3k == 1 & dat2$fhx_a_parent3l == 1] <- 1 # definite

fhx_a_parent3_aff

fhx_a_parent4_aff <- NULL

fhx_a_parent4_aff[dat2$fhx_a_parent4j == 1 | dat2$fhx_a_parent4j == 2 | dat2$fhx_a_parent4j == 3 | dat2$fhx_a_parent4j == 4] <- 0 # non-user, abstinent, social user

fhx_a_parent4_aff[(dat2$fhx_a_parent4j == 5 | dat2$fhx_a_parent4j == 2) & dat2$fhx_a_parent4k == 1 & dat2$fhx_a_parent4l == 0] <- 1 # probable

fhx_a_parent4_aff[(dat2$fhx_a_parent4j == 5 | dat2$fhx_a_parent4j == 2) & dat2$fhx_a_parent4k == 1 & dat2$fhx_a_parent4l == 1] <- 1 # definite

fhx_a_parent4_aff

fhx_a_parent5_aff <- NULL

fhx_a_parent5_aff[dat2$fhx_a_parent5j == 1 | dat2$fhx_a_parent5j == 2 | dat2$fhx_a_parent5j == 3 | dat2$fhx_a_parent5j == 4] <- 0 # non-user, abstinent, social user

fhx_a_parent5_aff[(dat2$fhx_a_parent5j == 5 | dat2$fhx_a_parent5j == 2) & dat2$fhx_a_parent5k == 1 & dat2$fhx_a_parent5l == 0] <- 1 # probable

fhx_a_parent5_aff[(dat2$fhx_a_parent5j == 5 | dat2$fhx_a_parent5j == 2) & dat2$fhx_a_parent5k == 1 & dat2$fhx_a_parent5l == 1] <- 1 # definite

fhx_a_parent5_aff

fhx_a_parent6_aff <- NULL

fhx_a_parent6_aff[dat2$fhx_a_parent6j == 1 | dat2$fhx_a_parent6j == 2 | dat2$fhx_a_parent6j == 3 | dat2$fhx_a_parent6j == 4] <- 0 # non-user, abstinent, social user

fhx_a_parent6_aff[(dat2$fhx_a_parent6j == 5 | dat2$fhx_a_parent6j == 2) & dat2$fhx_a_parent6k == 1 & dat2$fhx_a_parent6l == 0] <- 1 # probable

fhx_a_parent6_aff[(dat2$fhx_a_parent6j == 5 | dat2$fhx_a_parent6j == 2) & dat2$fhx_a_parent6k == 1 & dat2$fhx_a_parent6l == 1] <- 1 # definite

fhx_a_parent6_aff

fhx_a_gp1_aff <- NULL

fhx_a_gp1_aff[dat2$fhx_a_gp1j == 1 | dat2$fhx_a_gp1j == 2 | dat2$fhx_a_gp1j == 3 | dat2$fhx_a_gp1j == 4] <- 0 # non-user, abstinent, social user

fhx_a_gp1_aff[(dat2$fhx_a_gp1j == 5 | dat2$fhx_a_gp1j == 2) & dat2$fhx_a_gp1k == 1 & dat2$fhx_a_gp1l == 0] <- 1 # probable

fhx_a_gp1_aff[(dat2$fhx_a_gp1j == 5 | dat2$fhx_a_gp1j == 2) & dat2$fhx_a_gp1k == 1 & dat2$fhx_a_gp1l == 1] <- 1 # definite

fhx_a_gp1_aff

fhx_a_gp2_aff <- NULL

fhx_a_gp2_aff[dat2$fhx_a_gp2j == 1 | dat2$fhx_a_gp2j == 2 | dat2$fhx_a_gp2j == 3 | dat2$fhx_a_gp2j == 4] <- 0 # non-user, abstinent, social user

fhx_a_gp2_aff[(dat2$fhx_a_gp2j == 5 | dat2$fhx_a_gp2j == 2) & dat2$fhx_a_gp2k == 1 & dat2$fhx_a_gp2l == 0] <- 1 # probable

fhx_a_gp2_aff[(dat2$fhx_a_gp2j == 5 | dat2$fhx_a_gp2j == 2) & dat2$fhx_a_gp2k == 1 & dat2$fhx_a_gp2l == 1] <- 1 # definite

fhx_a_gp2_aff

fhx_a_gp3_aff <- NULL

fhx_a_gp3_aff[dat2$fhx_a_gp3j == 1 | dat2$fhx_a_gp3j == 2 | dat2$fhx_a_gp3j == 3 | dat2$fhx_a_gp3j == 4] <- 0 # non-user, abstinent, social user

fhx_a_gp3_aff[(dat2$fhx_a_gp3j == 5 | dat2$fhx_a_gp3j == 2) & dat2$fhx_a_gp3k == 1 & dat2$fhx_a_gp3l == 0] <- 1 # probable

fhx_a_gp3_aff[(dat2$fhx_a_gp3j == 5 | dat2$fhx_a_gp3j == 2) & dat2$fhx_a_gp3k == 1 & dat2$fhx_a_gp3l == 1] <- 1 # definite

fhx_a_gp3_aff

fhx_a_gp4_aff <- NULL

fhx_a_gp4_aff[dat2$fhx_a_gp4j == 1 | dat2$fhx_a_gp4j == 2 | dat2$fhx_a_gp4j == 3 | dat2$fhx_a_gp4j == 4] <- 0 # non-user, abstinent, social user

fhx_a_gp4_aff[(dat2$fhx_a_gp4j == 5 | dat2$fhx_a_gp4j == 2) & dat2$fhx_a_gp4k == 1 & dat2$fhx_a_gp4l == 0] <- 1 # probable

fhx_a_gp4_aff[(dat2$fhx_a_gp4j == 5 | dat2$fhx_a_gp4j == 2) & dat2$fhx_a_gp4k == 1 & dat2$fhx_a_gp4l == 1] <- 1 # definite

fhx_a_gp4_aff

fhx_a_gp5_aff <- NULL

fhx_a_gp5_aff[dat2$fhx_a_gp5j == 1 | dat2$fhx_a_gp5j == 2 | dat2$fhx_a_gp5j == 3 | dat2$fhx_a_gp5j == 4] <- 0 # non-user, abstinent, social user

fhx_a_gp5_aff[(dat2$fhx_a_gp5j == 5 | dat2$fhx_a_gp5j == 2) & dat2$fhx_a_gp5k == 1 & dat2$fhx_a_gp5l == 0] <- 1 # probable

fhx_a_gp5_aff[(dat2$fhx_a_gp5j == 5 | dat2$fhx_a_gp5j == 2) & dat2$fhx_a_gp5k == 1 & dat2$fhx_a_gp5l == 1] <- 1 # definite

fhx_a_gp5_aff

fhx_a_gp6_aff <- NULL

fhx_a_gp6_aff[dat2$fhx_a_gp6j == 1 | dat2$fhx_a_gp6j == 2 | dat2$fhx_a_gp6j == 3 | dat2$fhx_a_gp6j == 4] <- 0 # non-user, abstinent, social user

fhx_a_gp6_aff[(dat2$fhx_a_gp6j == 5 | dat2$fhx_a_gp6j == 2) & dat2$fhx_a_gp6k == 1 & dat2$fhx_a_gp6l == 0] <- 1 # probable

fhx_a_gp6_aff[(dat2$fhx_a_gp6j == 5 | dat2$fhx_a_gp6j == 2) & dat2$fhx_a_gp6k == 1 & dat2$fhx_a_gp6l == 1] <- 1 # definite

fhx_a_gp6_aff

fhx_a_gp7_aff <- NULL

fhx_a_gp7_aff[dat2$fhx_a_gp7j == 1 | dat2$fhx_a_gp7j == 2 | dat2$fhx_a_gp7j == 3 | dat2$fhx_a_gp7j == 4] <- 0 # non-user, abstinent, social user

fhx_a_gp7_aff[(dat2$fhx_a_gp7j == 5 | dat2$fhx_a_gp7j == 2) & dat2$fhx_a_gp7k == 1 & dat2$fhx_a_gp7l == 0] <- 1 # probable

fhx_a_gp7_aff[(dat2$fhx_a_gp7j == 5 | dat2$fhx_a_gp7j == 2) & dat2$fhx_a_gp7k == 1 & dat2$fhx_a_gp7l == 1] <- 1 # definite

fhx_a_gp7_aff

fhx_a_gp8_aff <- NULL

fhx_a_gp8_aff[dat2$fhx_a_gp8j == 1 | dat2$fhx_a_gp8j == 2 | dat2$fhx_a_gp8j == 3 | dat2$fhx_a_gp8j == 4] <- 0 # non-user, abstinent, social user

fhx_a_gp8_aff[(dat2$fhx_a_gp8j == 5 | dat2$fhx_a_gp8j == 2) & dat2$fhx_a_gp8k == 1 & dat2$fhx_a_gp8l == 0] <- 1 # probable

fhx_a_gp8_aff[(dat2$fhx_a_gp8j == 5 | dat2$fhx_a_gp8j == 2) & dat2$fhx_a_gp8k == 1 & dat2$fhx_a_gp8l == 1] <- 1 # definite

fhx_a_gp8_aff

fhx_a_gp9_aff <- NULL

fhx_a_gp9_aff[dat2$fhx_a_gp9j == 1 | dat2$fhx_a_gp9j == 2 | dat2$fhx_a_gp9j == 3 | dat2$fhx_a_gp9j == 4] <- 0 # non-user, abstinent, social user

fhx_a_gp9_aff[(dat2$fhx_a_gp9j == 5 | dat2$fhx_a_gp9j == 2) & dat2$fhx_a_gp9k == 1 & dat2$fhx_a_gp9l == 0] <- 1 # probable

fhx_a_gp9_aff[(dat2$fhx_a_gp9j == 5 | dat2$fhx_a_gp9j == 2) & dat2$fhx_a_gp9k == 1 & dat2$fhx_a_gp9l == 1] <- 1 # definite

fhx_a_gp9_aff

fhx_a_gp10_aff <- NULL

fhx_a_gp10_aff[dat2$fhx_a_gp10j == 1 | dat2$fhx_a_gp10j == 2 | dat2$fhx_a_gp10j == 3 | dat2$fhx_a_gp10j == 4] <- 0 # non-user, abstinent, social user

fhx_a_gp10_aff[(dat2$fhx_a_gp10j == 5 | dat2$fhx_a_gp10j == 2) & dat2$fhx_a_gp10k == 1 & dat2$fhx_a_gp10l == 0] <- 1 # probable

fhx_a_gp10_aff[(dat2$fhx_a_gp10j == 5 | dat2$fhx_a_gp10j == 2) & dat2$fhx_a_gp10k == 1 & dat2$fhx_a_gp10l == 1] <- 1 # definite

fhx_a_gp10_aff

fhx_a_aunt1_aff <- NULL

fhx_a_aunt1_aff[dat2$fhx_a_aunt1j == 1 | dat2$fhx_a_aunt1j == 2 | dat2$fhx_a_aunt1j == 3 | dat2$fhx_a_aunt1j == 4] <- 0 # non-user, abstinent, social user

fhx_a_aunt1_aff[(dat2$fhx_a_aunt1j == 5 | dat2$fhx_a_aunt1j == 2) & dat2$fhx_a_aunt1k == 1 & dat2$fhx_a_aunt1l == 0] <- 1 # probable

fhx_a_aunt1_aff[(dat2$fhx_a_aunt1j == 5 | dat2$fhx_a_aunt1j == 2) & dat2$fhx_a_aunt1k == 1 & dat2$fhx_a_aunt1l == 1] <- 1 # definite

fhx_a_aunt1_aff

fhx_a_aunt2_aff <- NULL

fhx_a_aunt2_aff[dat2$fhx_a_aunt2j == 1 | dat2$fhx_a_aunt2j == 2 | dat2$fhx_a_aunt2j == 3 | dat2$fhx_a_aunt2j == 4] <- 0 # non-user, abstinent, social user

fhx_a_aunt2_aff[(dat2$fhx_a_aunt2j == 5 | dat2$fhx_a_aunt2j == 2) & dat2$fhx_a_aunt2k == 1 & dat2$fhx_a_aunt2l == 0] <- 1 # probable

fhx_a_aunt2_aff[(dat2$fhx_a_aunt2j == 5 | dat2$fhx_a_aunt2j == 2) & dat2$fhx_a_aunt2k == 1 & dat2$fhx_a_aunt2l == 1] <- 1 # definite

fhx_a_aunt2_aff

fhx_a_aunt3_aff <- NULL

fhx_a_aunt3_aff[dat2$fhx_a_aunt3j == 1 | dat2$fhx_a_aunt3j == 2 | dat2$fhx_a_aunt3j == 3 | dat2$fhx_a_aunt3j == 4] <- 0 # non-user, abstinent, social user

fhx_a_aunt3_aff[(dat2$fhx_a_aunt3j == 5 | dat2$fhx_a_aunt3j == 2) & dat2$fhx_a_aunt3k == 1 & dat2$fhx_a_aunt3l == 0] <- 1 # probable

fhx_a_aunt3_aff[(dat2$fhx_a_aunt3j == 5 | dat2$fhx_a_aunt3j == 2) & dat2$fhx_a_aunt3k == 1 & dat2$fhx_a_aunt3l == 1] <- 1 # definite

fhx_a_aunt3_aff

fhx_a_aunt4_aff <- NULL

fhx_a_aunt4_aff[dat2$fhx_a_aunt4j == 1 | dat2$fhx_a_aunt4j == 2 | dat2$fhx_a_aunt4j == 3 | dat2$fhx_a_aunt4j == 4] <- 0 # non-user, abstinent, social user

fhx_a_aunt4_aff[(dat2$fhx_a_aunt4j == 5 | dat2$fhx_a_aunt4j == 2) & dat2$fhx_a_aunt4k == 1 & dat2$fhx_a_aunt4l == 0] <- 1 # probable

fhx_a_aunt4_aff[(dat2$fhx_a_aunt4j == 5 | dat2$fhx_a_aunt4j == 2) & dat2$fhx_a_aunt4k == 1 & dat2$fhx_a_aunt4l == 1] <- 1 # definite

fhx_a_aunt4_aff

fhx_a_aunt5_aff <- NULL

fhx_a_aunt5_aff[dat2$fhx_a_aunt5j == 1 | dat2$fhx_a_aunt5j == 2 | dat2$fhx_a_aunt5j == 3 | dat2$fhx_a_aunt5j == 4] <- 0 # non-user, abstinent, social user

fhx_a_aunt5_aff[(dat2$fhx_a_aunt5j == 5 | dat2$fhx_a_aunt5j == 2) & dat2$fhx_a_aunt5k == 1 & dat2$fhx_a_aunt5l == 0] <- 1 # probable

fhx_a_aunt5_aff[(dat2$fhx_a_aunt5j == 5 | dat2$fhx_a_aunt5j == 2) & dat2$fhx_a_aunt5k == 1 & dat2$fhx_a_aunt5l == 1] <- 1 # definite

fhx_a_aunt5_aff

fhx_a_aunt6_aff <- NULL

fhx_a_aunt6_aff[dat2$fhx_a_aunt6j == 1 | dat2$fhx_a_aunt6j == 2 | dat2$fhx_a_aunt6j == 3 | dat2$fhx_a_aunt6j == 4] <- 0 # non-user, abstinent, social user

fhx_a_aunt6_aff[(dat2$fhx_a_aunt6j == 5 | dat2$fhx_a_aunt6j == 2) & dat2$fhx_a_aunt6k == 1 & dat2$fhx_a_aunt6l == 0] <- 1 # probable

fhx_a_aunt6_aff[(dat2$fhx_a_aunt6j == 5 | dat2$fhx_a_aunt6j == 2) & dat2$fhx_a_aunt6k == 1 & dat2$fhx_a_aunt6l == 1] <- 1 # definite

fhx_a_aunt6_aff

fhx_a_aunt7_aff <- NULL

fhx_a_aunt7_aff[dat2$fhx_a_aunt7j == 1 | dat2$fhx_a_aunt7j == 2 | dat2$fhx_a_aunt7j == 3 | dat2$fhx_a_aunt7j == 4] <- 0 # non-user, abstinent, social user

fhx_a_aunt7_aff[(dat2$fhx_a_aunt7j == 5 | dat2$fhx_a_aunt7j == 2) & dat2$fhx_a_aunt7k == 1 & dat2$fhx_a_aunt7l == 0] <- 1 # probable

fhx_a_aunt7_aff[(dat2$fhx_a_aunt7j == 5 | dat2$fhx_a_aunt7j == 2) & dat2$fhx_a_aunt7k == 1 & dat2$fhx_a_aunt7l == 1] <- 1 # definite

fhx_a_aunt7_aff

fhx_a_aunt8_aff <- NULL

fhx_a_aunt8_aff[dat2$fhx_a_aunt8j == 1 | dat2$fhx_a_aunt8j == 2 | dat2$fhx_a_aunt8j == 3 | dat2$fhx_a_aunt8j == 4] <- 0 # non-user, abstinent, social user

fhx_a_aunt8_aff[(dat2$fhx_a_aunt8j == 5 | dat2$fhx_a_aunt8j == 2) & dat2$fhx_a_aunt8k == 1 & dat2$fhx_a_aunt8l == 0] <- 1 # probable

fhx_a_aunt8_aff[(dat2$fhx_a_aunt8j == 5 | dat2$fhx_a_aunt8j == 2) & dat2$fhx_a_aunt8k == 1 & dat2$fhx_a_aunt8l == 1] <- 1 # definite

fhx_a_aunt8_aff

fhx_a_aunt9_aff <- NULL

fhx_a_aunt9_aff[dat2$fhx_a_aunt9j == 1 | dat2$fhx_a_aunt9j == 2 | dat2$fhx_a_aunt9j == 3 | dat2$fhx_a_aunt9j == 4] <- 0 # non-user, abstinent, social user

fhx_a_aunt9_aff[(dat2$fhx_a_aunt9j == 5 | dat2$fhx_a_aunt9j == 2) & dat2$fhx_a_aunt9k == 1 & dat2$fhx_a_aunt9l == 0] <- 1 # probable

fhx_a_aunt9_aff[(dat2$fhx_a_aunt9j == 5 | dat2$fhx_a_aunt9j == 2) & dat2$fhx_a_aunt9k == 1 & dat2$fhx_a_aunt9l == 1] <- 1 # definite

fhx_a_aunt9_aff

fhx_a_aunt10_aff <- NULL

fhx_a_aunt10_aff[dat2$fhx_a_aunt10j == 1 | dat2$fhx_a_aunt10j == 2 | dat2$fhx_a_aunt10j == 3 | dat2$fhx_a_aunt10j == 4] <- 0 # non-user, abstinent, social user

fhx_a_aunt10_aff[(dat2$fhx_a_aunt10j == 5 | dat2$fhx_a_aunt10j == 2) & dat2$fhx_a_aunt10k == 1 & dat2$fhx_a_aunt10l == 0] <- 1 # probable

fhx_a_aunt10_aff[(dat2$fhx_a_aunt10j == 5 | dat2$fhx_a_aunt10j == 2) & dat2$fhx_a_aunt10k == 1 & dat2$fhx_a_aunt10l == 1] <- 1 # definite

fhx_a_aunt10_aff

fhx_a_uncle1_aff <- NULL

fhx_a_uncle1_aff[dat2$fhx_a_uncle1j == 1 | dat2$fhx_a_uncle1j == 2 | dat2$fhx_a_uncle1j == 3 | dat2$fhx_a_uncle1j == 4] <- 0 # non-user, abstinent, social user

fhx_a_uncle1_aff[(dat2$fhx_a_uncle1j == 5 | dat2$fhx_a_uncle1j == 2) & dat2$fhx_a_uncle1k == 1 & dat2$fhx_a_uncle1l == 0] <- 1 # probable

fhx_a_uncle1_aff[(dat2$fhx_a_uncle1j == 5 | dat2$fhx_a_uncle1j == 2) & dat2$fhx_a_uncle1k == 1 & dat2$fhx_a_uncle1l == 1] <- 1 # definite

fhx_a_uncle1_aff

fhx_a_uncle2_aff <- NULL

fhx_a_uncle2_aff[dat2$fhx_a_uncle2j == 1 | dat2$fhx_a_uncle2j == 2 | dat2$fhx_a_uncle2j == 3 | dat2$fhx_a_uncle2j == 4] <- 0 # non-user, abstinent, social user

fhx_a_uncle2_aff[(dat2$fhx_a_uncle2j == 5 | dat2$fhx_a_uncle2j == 2) & dat2$fhx_a_uncle2k == 1 & dat2$fhx_a_uncle2l == 0] <- 1 # probable

fhx_a_uncle2_aff[(dat2$fhx_a_uncle2j == 5 | dat2$fhx_a_uncle2j == 2) & dat2$fhx_a_uncle2k == 1 & dat2$fhx_a_uncle2l == 1] <- 1 # definite

fhx_a_uncle2_aff

fhx_a_uncle3_aff <- NULL

fhx_a_uncle3_aff[dat2$fhx_a_uncle3j == 1 | dat2$fhx_a_uncle3j == 2 | dat2$fhx_a_uncle3j == 3 | dat2$fhx_a_uncle3j == 4] <- 0 # non-user, abstinent, social user

fhx_a_uncle3_aff[(dat2$fhx_a_uncle3j == 5 | dat2$fhx_a_uncle3j == 2) & dat2$fhx_a_uncle3k == 1 & dat2$fhx_a_uncle3l == 0] <- 1 # probable

fhx_a_uncle3_aff[(dat2$fhx_a_uncle3j == 5 | dat2$fhx_a_uncle3j == 2) & dat2$fhx_a_uncle3k == 1 & dat2$fhx_a_uncle3l == 1] <- 1 # definite

fhx_a_uncle3_aff

fhx_a_uncle4_aff <- NULL

fhx_a_uncle4_aff[dat2$fhx_a_uncle4j == 1 | dat2$fhx_a_uncle4j == 2 | dat2$fhx_a_uncle4j == 3 | dat2$fhx_a_uncle4j == 4] <- 0 # non-user, abstinent, social user

fhx_a_uncle4_aff[(dat2$fhx_a_uncle4j == 5 | dat2$fhx_a_uncle4j == 2) & dat2$fhx_a_uncle4k == 1 & dat2$fhx_a_uncle4l == 0] <- 1 # probable

fhx_a_uncle4_aff[(dat2$fhx_a_uncle4j == 5 | dat2$fhx_a_uncle4j == 2) & dat2$fhx_a_uncle4k == 1 & dat2$fhx_a_uncle4l == 1] <- 1 # definite

fhx_a_uncle4_aff

fhx_a_uncle5_aff <- NULL

fhx_a_uncle5_aff[dat2$fhx_a_uncle5j == 1 | dat2$fhx_a_uncle5j == 2 | dat2$fhx_a_uncle5j == 3 | dat2$fhx_a_uncle5j == 4] <- 0 # non-user, abstinent, social user

fhx_a_uncle5_aff[(dat2$fhx_a_uncle5j == 5 | dat2$fhx_a_uncle5j == 2) & dat2$fhx_a_uncle5k == 1 & dat2$fhx_a_uncle5l == 0] <- 1 # probable

fhx_a_uncle5_aff[(dat2$fhx_a_uncle5j == 5 | dat2$fhx_a_uncle5j == 2) & dat2$fhx_a_uncle5k == 1 & dat2$fhx_a_uncle5l == 1] <- 1 # definite

fhx_a_uncle5_aff

fhx_a_uncle6_aff <- NULL

fhx_a_uncle6_aff[dat2$fhx_a_uncle6j == 1 | dat2$fhx_a_uncle6j == 2 | dat2$fhx_a_uncle6j == 3 | dat2$fhx_a_uncle6j == 4] <- 0 # non-user, abstinent, social user

fhx_a_uncle6_aff[(dat2$fhx_a_uncle6j == 5 | dat2$fhx_a_uncle6j == 2) & dat2$fhx_a_uncle6k == 1 & dat2$fhx_a_uncle6l == 0] <- 1 # probable

fhx_a_uncle6_aff[(dat2$fhx_a_uncle6j == 5 | dat2$fhx_a_uncle6j == 2) & dat2$fhx_a_uncle6k == 1 & dat2$fhx_a_uncle6l == 1] <- 1 # definite

fhx_a_uncle6_aff

fhx_a_uncle7_aff <- NULL

fhx_a_uncle7_aff[dat2$fhx_a_uncle7j == 1 | dat2$fhx_a_uncle7j == 2 | dat2$fhx_a_uncle7j == 3 | dat2$fhx_a_uncle7j == 4] <- 0 # non-user, abstinent, social user

fhx_a_uncle7_aff[(dat2$fhx_a_uncle7j == 5 | dat2$fhx_a_uncle7j == 2) & dat2$fhx_a_uncle7k == 1 & dat2$fhx_a_uncle7l == 0] <- 1 # probable

fhx_a_uncle7_aff[(dat2$fhx_a_uncle7j == 5 | dat2$fhx_a_uncle7j == 2) & dat2$fhx_a_uncle7k == 1 & dat2$fhx_a_uncle7l == 1] <- 1 # definite

fhx_a_uncle7_aff

fhx_a_uncle8_aff <- NULL

fhx_a_uncle8_aff[dat2$fhx_a_uncle8j == 1 | dat2$fhx_a_uncle8j == 2 | dat2$fhx_a_uncle8j == 3 | dat2$fhx_a_uncle8j == 4] <- 0 # non-user, abstinent, social user

fhx_a_uncle8_aff[(dat2$fhx_a_uncle8j == 5 | dat2$fhx_a_uncle8j == 2) & dat2$fhx_a_uncle8k == 1 & dat2$fhx_a_uncle8l == 0] <- 1 # probable

fhx_a_uncle8_aff[(dat2$fhx_a_uncle8j == 5 | dat2$fhx_a_uncle8j == 2) & dat2$fhx_a_uncle8k == 1 & dat2$fhx_a_uncle8l == 1] <- 1 # definite

fhx_a_uncle8_aff

fhx_a_uncle9_aff <- NULL

fhx_a_uncle9_aff[dat2$fhx_a_uncle9j == 1 | dat2$fhx_a_uncle9j == 2 | dat2$fhx_a_uncle9j == 3 | dat2$fhx_a_uncle9j == 4] <- 0 # non-user, abstinent, social user

fhx_a_uncle9_aff[(dat2$fhx_a_uncle9j == 5 | dat2$fhx_a_uncle9j == 2) & dat2$fhx_a_uncle9k == 1 & dat2$fhx_a_uncle9l == 0] <- 1 # probable

fhx_a_uncle9_aff[(dat2$fhx_a_uncle9j == 5 | dat2$fhx_a_uncle9j == 2) & dat2$fhx_a_uncle9k == 1 & dat2$fhx_a_uncle9l == 1] <- 1 # definite

fhx_a_uncle9_aff

fhx_a_uncle10_aff <- NULL

fhx_a_uncle10_aff[dat2$fhx_a_uncle10j == 1 | dat2$fhx_a_uncle10j == 2 | dat2$fhx_a_uncle10j == 3 | dat2$fhx_a_uncle10j == 4] <- 0 # non-user, abstinent, social user

fhx_a_uncle10_aff[(dat2$fhx_a_uncle10j == 5 | dat2$fhx_a_uncle10j == 2) & dat2$fhx_a_uncle10k == 1 & dat2$fhx_a_uncle10l == 0] <- 1 # probable

fhx_a_uncle10_aff[(dat2$fhx_a_uncle10j == 5 | dat2$fhx_a_uncle10j == 2) & dat2$fhx_a_uncle10k == 1 & dat2$fhx_a_uncle10l == 1] <- 1 # definite

fhx_a_uncle10_aff

# Calculate W, or relateness

rel <- c(dat2$fhx_a_sib1_rel, dat2$fhx_a_sib2_rel, dat2$fhx_a_sib3_rel, dat2$fhx_a_sib4_rel, dat2$fhx_a_sib5_rel,

dat2$fhx_a_sib6_rel, dat2$fhx_a_sib7_rel, dat2$fhx_a_sib8_rel, dat2$fhx_a_sib9_rel, dat2$fhx_a_sib10_rel,

dat2$fhx_a_parent1_rel, dat2$fhx_a_parent2_rel, dat2$fhx_a_parent3_rel, dat2$fhx_a_parent4_rel, dat2$fhx_a_parent5_rel, dat2$fhx_a_parent6_rel,

dat2$fhx_a_gp1_rel, dat2$fhx_a_gp2_rel, dat2$fhx_a_gp3_rel, dat2$fhx_a_gp4_rel, dat2$fhx_a_gp5_rel,

dat2$fhx_a_gp6_rel, dat2$fhx_a_gp7_rel, dat2$fhx_a_gp8_rel, dat2$fhx_a_gp9_rel, dat2$fhx_a_gp10_rel,

dat2$fhx_a_aunt1_rel, dat2$fhx_a_aunt2_rel, dat2$fhx_a_aunt3_rel, dat2$fhx_a_aunt4_rel, dat2$fhx_a_aunt5_rel,

dat2$fhx_a_aunt6_rel, dat2$fhx_a_aunt7_rel, dat2$fhx_a_aunt8_rel, dat2$fhx_a_aunt9_rel, dat2$fhx_a_aunt10_rel,

dat2$fhx_a_uncle1_rel, dat2$fhx_a_uncle2_rel, dat2$fhx_a_uncle3_rel, dat2$fhx_a_uncle4_rel, dat2$fhx_a_uncle5_rel,

dat2$fhx_a_uncle6_rel, dat2$fhx_a_uncle7_rel, dat2$fhx_a_uncle8_rel, dat2$fhx_a_uncle9_rel, dat2$fhx_a_uncle10_rel)

rel[rel==1] <- 0.5

rel[rel==2] <- 0.25

rel[rel==3] <- NA # don't want 0.125 people; only focus on 1st and 2nd; only included it as an option in case there are weird relatedness

rel[rel==4] <- 0 # married, adopted, etc.

rel

#Calculate sum(W), or relatedness sum

rel_sum <- sum(rel, na.rm = TRUE)

rel_sum

# Calculate A, or affectedness

aff <- c(fhx_a_sib1_aff, fhx_a_sib2_aff, fhx_a_sib3_aff, fhx_a_sib4_aff, fhx_a_sib5_aff,

fhx_a_sib6_aff, fhx_a_sib7_aff, fhx_a_sib8_aff, fhx_a_sib9_aff, fhx_a_sib10_aff,

fhx_a_parent1_aff, fhx_a_parent2_aff, fhx_a_parent3_aff, fhx_a_parent4_aff, fhx_a_parent5_aff, fhx_a_parent6_aff,

fhx_a_gp1_aff, fhx_a_gp2_aff, fhx_a_gp3_aff, fhx_a_gp4_aff, fhx_a_gp5_aff,

fhx_a_gp6_aff, fhx_a_gp7_aff, fhx_a_gp8_aff, fhx_a_gp9_aff, fhx_a_gp10_aff,

fhx_a_aunt1_aff, fhx_a_aunt2_aff, fhx_a_aunt3_aff, fhx_a_aunt4_aff, fhx_a_aunt5_aff,

fhx_a_aunt6_aff, fhx_a_aunt7_aff, fhx_a_aunt8_aff, fhx_a_aunt9_aff, fhx_a_aunt10_aff,

fhx_a_uncle1_aff, fhx_a_uncle2_aff, fhx_a_uncle3_aff, fhx_a_uncle4_aff, fhx_a_uncle5_aff,

fhx_a_uncle6_aff, fhx_a_uncle7_aff, fhx_a_uncle8_aff, fhx_a_uncle9_aff, fhx_a_uncle10_aff)

aff

# Multiply relatedness and affectedness; sum; divide by relatedness sum to scale it

famden_alc <- (sum(rel*aff, na.rm = TRUE)) / rel_sum

famden_alc
